# Comparison of linear model and artificial neural network using antler beam diameter and length of white-tailed deer (*Odocoileus virginianus*) dataset

**DOI:** 10.1371/journal.pone.0212545

**Published:** 2019-02-22

**Authors:** Sunday O. Peters, Mahmut Sinecen, George R. Gallagher, Lauren A. Pebworth, Suleima Jacob, Jason S. Hatfield, Kadir Kizilkaya

**Affiliations:** 1 Department of Animal Science, School of Mathematical and Natural Sciences, Berry College, Mount Berry, Georgia, United States of America; 2 Department of Animal and Dairy Science, University of Georgia, Athens, Georgia, United States of America; 3 Department of Computer Engineering, Faculty of Engineering, Aydin Adnan Menderes University, Aydin, Turkey; 4 Department of Animal Science, Faculty of Agriculture, Aydin Adnan Menderes University, Aydin, Turkey; University of Otago, NEW ZEALAND

## Abstract

Evaluation of harvest data remains one of the most important sources of information in the development of strategies to manage regional populations of white-tailed deer. While descriptive statistics and simple linear models are utilized extensively, the use of artificial neural networks for this type of data analyses is unexplored. Linear model was compared to Artificial Neural Networks (ANN) models with Levenberg–Marquardt (L-M), Bayesian Regularization (BR) and Scaled Conjugate Gradient (SCG) learning algorithms, to evaluate the relative accuracy in predicting antler beam diameter and length using age and dressed body weight in white-tailed deer. Data utilized for this study were obtained from male animals harvested by hunters between 1977–2009 at the Berry College Wildlife Management Area. Metrics for evaluating model performance indicated that linear and ANN models resulted in close match and good agreement between predicted and observed values and thus good performance for all models. However, metrics values of Mean Absolute Error and Root Mean Squared Error for linear model and the ANN-BR model indicated smaller error and lower deviation relative to the mean values of antler beam diameter and length in comparison to other ANN models, demonstrating better agreement of the predicted and observed values of antler beam diameter and length. ANN-SCG model resulted in the highest error within the models. Overall, metrics for evaluating model performance from the ANN model with BR learning algorithm and linear model indicated better agreement of the predicted and observed values of antler beam diameter and length. Results of this study suggest the use of ANN generated results that are comparable to Linear Models of harvest data to aid in the development of strategies to manage white-tailed deer.

## Introduction

White-tailed deer (*Odocoileus virginianus*) (WTD) are among the largest herbivore ungulates in forested ecosystems of the eastern United States [1]. Numbers of this animal represents a significant economic impact. Hunting in North America contributed an estimated $38.3 billion annually to the economy of the United States [2]. In 2001, an estimated 10.3 million hunters harvested deer and in 2006 approximately 10.7 million hunters harvested 6.2 million deer in the United State of America [1, 3]. In contrast, overabundance of WTD can lead to human-wildlife conflict. Based upon the National crash data base (2001–2005) approximately 300,000 wildlife-vehicle collisions per year are reported, with a vast majority of those events involving deer [4]. Economic damage of wildlife-vehicle collisions was reported to exceed $8 billion/year [4] Annual damage to vegetable and grain crops in the northeastern United States was estimated to exceed $168 million [5]. Therefore, it becomes critical to manage the population of WTD to meet objectives in a given environment. One of the most important sources of information utilized to develop WTD management plans is availability of annual harvest records for a given area. Information related to animal physical characteristics provide critical information about the number and overall health status of the regional deer herd. These records can assist managers in determining the appropriate management strategies to achieve defined objectives.

The relationships among antler characteristics, age and field-dressed weight and predictions of these traits in deer populations have been studied by using linear models [6, 7]. Significant literature exists related to the use of body measurement, such as antler size, to predict age, as well as correlation between antler size and body weight. While antler size and body mass are common measurements recorded from harvest data, these two characteristics are often evaluated separately. Studies typically focus on correlations between specific factors on either antler traits or body size [3, 8–13]. For example, Hewitt et al. [14] reported a weak positive relationship (Pearson correlation coefficient = 0.37), between yearling antler size and girth size. The use of this type of information is reported to be used by managers to evaluate the results of harvest criteria to develop future management plans [3, 14–16].

While the concept of Artificial Neural Network (ANN) for statistical analysis was initiated as early as 1911, most significant developments of application have occurred in the past 50 years [17, 18]. A review of current literature indicates ANNs have been used successfully in pattern recognition, classification, aspects of prediction and forecasting, modeling problems in medicine and engineering, and agricultural applications [19–23]. The concept of ANN for statistical analysis was inspired by the potential to mimic organization and communication pathways found in the nervous systems of organisms. The most common methodology for utilization of ANN is to divide the dataset into training (learning) and test datasets. The training dataset is used to train the network and to determine the best ANN architecture by choosing the number of neurons in the hidden layer based on a learning algorithm such as Levenberg–Marquardt (L-M), Bayesian Regularization (BR) and Scaled Conjugate Gradient (SCG) backpropagation. The test dataset is used to test network performance and to validate the model based on new and unseen datasets [22–24].

Therefore, our objective was to compare the performances of a typical linear model to ANN with three different learning algorithms of L-M, BR and SCG backpropagation on the predictions of antler beam diameter and length using age and field-dressed weights of harvested WTD.

## Materials and methods

### Deer population and study area

Access to annual deer harvest data (1971–2014) collected from the Berry College Wildlife Management Area (BCWMA) was granted by scientists working with the Georgia Department of Natural Resources (GDNR). We did not kill these animals and so we did not need IACUC approval for this research.

The Berry College campus, located in Northwest Georgia, USA (34.2904° N, 85.1892° W) consists of 11,340 ha, with approximately 1,215 ha maintained as the Berry College Wildlife Refuge. In an effort to provide public access to areas available for hunting, the state of Georgia, through the GDNR–Wildlife Resources Division, leases large tracts of land on an annual basis from private land holders. These areas are designated as Wildlife Management Areas. The Berry College Wildlife Management Area (BCWMA) has been in existence since 1971 consisting of an average of 7,200 ha.

The BCWMA is within the Ridge and Valley physiographic province with elevations ranging from 172 m to 518 m [25]. The BCWMA consisted of mixed forest dominated by pine (*Pinus* spp.), oaks (*Quercus* spp.), hickories (*Carya* spp.), openings of native grasses such as Big bluestem (*Andropogon gerardi*), Little bluestem (*Schizachyrium scoparium*), Indian grass (*Sorghastrum nutans*), Eastern game grass (*Tripsacum dactyloides*), Broomsedge (*Andropogon virginicus*) and Switchgrass (*Panicum* spp) and wetland areas at lower elevations. Typical precipitation in the area was > 130 cm/year. The BCWMA had a deer population density at 12 deer/km^2^ [26].

Wildlife Management Areas are managed by the GDNR in cooperation with the land holder. As such, hunting access and seasons are typically different than the general state regulations. While changes in the BCWMA related to hunting of WTD have occurred since 1971 to the current year, in general there have been 2–4, 2-day to 4-day firearm hunts offered in October–December, annually. A quota system to regulate the number of hunters permitted for each hunt has also occurred. All animals harvested during the firearm hunts are required to be transported to a check station managed by the GDNR. The GDNR have been collecting data including age and sex of animals, field dressed weight, antler characteristics of males as well as information relating to hunter demographics since 1971-current year. A subset (1977–2008) of this data was utilized for this study.

### Training and testing datasets

BCWMA deer harvest dataset includes the variables of year and month of harvest (September, October, November, December), field-dressed weight (weight of animal without internal organs, (kg)), antler beam diameter (estimated diameter of the main beam typically obtained immediately cranial to the burr of the antler, (cm)), and length of antler (measured from the burr to the tip of the main beam, (cm)) between 1971 and 2014. The WTD harvest dataset were created by using 2,899 observations from male deer between 1977 and 2008 after cleaning missing observations of antler beam diameter and length in the BCWMA deer harvest data.

The WTD harvest dataset for the statistical analysis and model comparison was split into two subsets: training and testing datasets. The training dataset was created by using randomly 2,543 observations (88%) of the WTD harvest dataset for training in the development of model and parameter estimation. Table 1 shows distribution of training WTD harvest data across year and month. As seen in Table 1, the distribution of 2,543 observations was unbalanced and sparse across year and month. There were no observations between 1978 and 1987 for the months of 11 and 12, and between 1996 and 2007 for the months of 9 and 10, which makes it not possible to add the interaction term between year and month into the linear model. Due to variations between years related to particular month a given firearms hunt was available, the interaction term between year and month was not included into the linear model.

**Table 1:** The distribution of training white-tailed deer harvest dataset across year and month.

Year	Month				Year	Month			
	9	10	11	12		9	10	11	12
1977	3	65	28	0	1993	0	47	44	0
1978	0	65	0	0	1994	0	63	25	1
1979	0	37	0	0	1995	0	42	32	1
1980	3	46	0	0	1996	0	0	118	0
1981	5	48	0	0	1997	0	0	106	0
1982	5	50	0	0	1998	0	0	80	21
1983	7	34	0	0	1999	0	0	85	13
1984	2	45	0	0	2000	0	0	53	9
1985	8	62	0	0	2001	0	0	85	8
1986	5	81	0	0	2002	0	0	76	11
1987	0	63	0	40	2003	0	0	65	24
1988	4	12	103	0	2004	0	0	42	20
1989	0	18	53	14	2005	0	0	49	9
1990	3	4	71	17	2006	0	0	66	10
1991	0	43	47	0	2007	0	9	52	2
1992	0	72	38	0	2008	0	0	58	16

The testing WTD dataset was 12% of the deer harvest dataset, independent of training dataset was used to validate the model. There were 356 observations within the testing dataset representing all months and years (Table 2).

**Table 2:** The distribution of testing white-tailed deer harvest dataset across year and month.

Year	Month				Year	Month			
	9	10	11	12		9	10	11	12
1977	0	12	2	0	1993	0	13	5	0
1978	0	9	0	0	1994	0	12	3	0
1979	0	6	0	0	1995	0	7	7	0
1980	1	7	0	0	1996	0	0	15	0
1981	1	9	0	0	1997	0	0	10	0
1982	1	7	0	0	1998	0	0	11	2
1983	0	9	0	0	1999	0	0	15	2
1984	1	5	0	0	2000	0	0	10	1
1985	1	10	0	0	2001	0	0	16	0
1986	1	5	0	0	2002	1	0	8	2
1987	0	6	0	4	2003	0	0	11	5
1988	0	0	15	0	2004	0	0	3	3
1989	0	0	9	3	2005	0	0	11	1
1990	0	0	5	2	2006	0	0	8	1
1991	0	8	7	0	2007	0	1	5	1
1992	0	3	5	0	2008	0	0	9	3

### Statistical analysis

#### Linear model

The linear model in Eq 1 was applied for the statistical analysis of antler beam diameter and length:

$$Y_{ijk}=\mu +\alpha_{i}+\tau_{j}+\beta_{1}(x_{1ijk}-{\bar{x}}_{1})+\beta_{2}(x_{2ijk}-{\bar{x}}_{2})+e_{ijk}$$ (1)

where *Y*_*ijk*_ is the response variable for antler beam diameter or length; *μ* is the overall mean for antler beam diameter or length; *α*_*i*_ is the *i*^th^ hunting year effect (*i* = 1977, 1978, …, 2008); *τ*_*j*_ is the *j*^th^ hunting month effect (*j* = 9, 10, 11, 12); *β*_1_ and *β*_2_ are the regression coefficients of age (*x*_1_) and field-dressed weight (*x*_2_) of hunted WTD; ${\bar{x}}_{1}$ and ${\bar{x}}_{2}$ are the averages of age (*x*_1_) and field-dressed weight (*x*_2_) and *e*_*ijk*_ is the normally distributed error term with mean zero and variance *σ*^2^.

The lm package for R [27] was used to fit the linear model shown in Eq 1. Statistical significance of model terms was determined with F-tests. After significant effects of factors were identified, differences between least square means of hunting years and months were considered significant at *p*<0.05 (2-tailed) based on the type I error rate.

#### Artificial neural networks

ANN is comprised of neurons as data processing units which are connected via adjustable weights (*w*_*i*_). Neurons are arranged based on their functions in layers, an input layer, hidden layer(s), and an output layer. In the input layer, each neuron is designated to each input variable (*x*_*i*_). The training or learning process for pattern recognition, classification or prediction in ANN is carried out by comparing the ANN simulated output values $\left({\hat{Y}}_{i}\right)$ to the observed (actual) values (*Y*_*i*_) and calculating a prediction error using training dataset. The error $\left({\hat{Y}}_{i}-Y_{i}\right)$ is then back propagated through the network and weights are adjusted as the network attempts to decrease the prediction error by optimizing the weights that contribute most to the error by using a learning algorithm [23]. There are many types of learning algorithms in the literature [28–30]. The objective of every learning algorithm is to reduce the global error by adjusting the weights and biases in the ANN procedure. However, it is very difficult to know which learning algorithm will be more efficient for a given problem. In this study, L-M, BR and SCG backpropagation algorithms were used to determine the ANN algorithm providing faster learning and producing better estimates in the analysis of antler beam diameter and length.

Multi-Layer Perceptrons (MLP) are the simplest and most commonly used ANN architecture due to their structural flexibility, good representational capabilities and large number of programmable algorithms [31, 32]. The feedforward Multi-Layer Perceptron Artificial Neural Network (MLPANN) was proposed for this study and composed of many interconnected neurons which are grouped into an input layer, an output layer and an intermediate or hidden layer. An MLPANN is a fully connected network since every neuron is connected to all neurons of the next layer.

**Fig 1:**
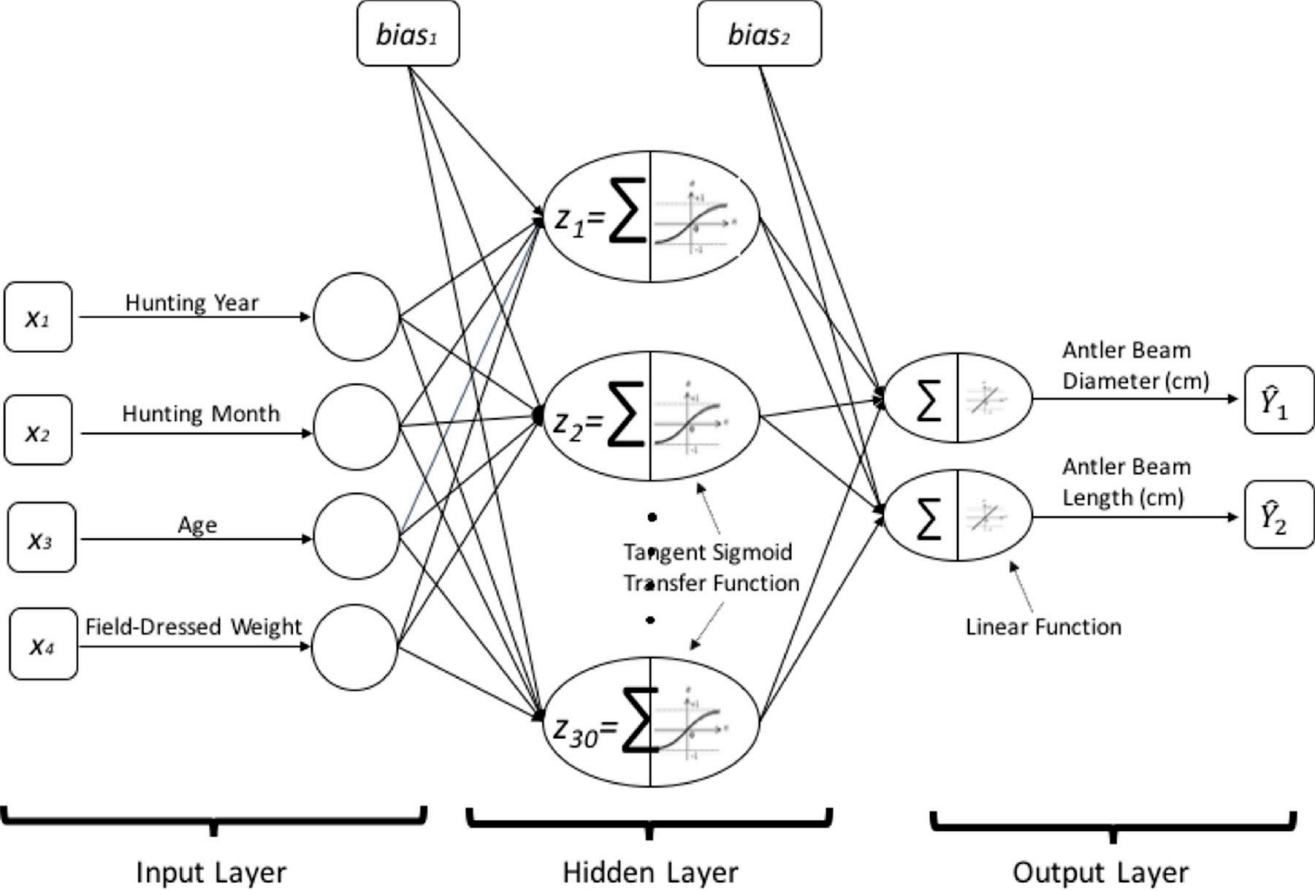
Artificial neural networks architecture for white-tailed deer (*Odocoileus virginianus*) dataset.

In the present study utilizing the MATLAB Neural Network Toolbox, the feedforward MLPANN model was structured with three layers including an input, one hidden and an output layer [33]. Graphical representation of the proposed MLPANN model is shown in Fig 1. As seen in Fig 1, in the MLPANN model, independent variables of the hunting year and month, age and field-dressed weight in the input layer represent the four input neurons. Also, dependent variables of antler beam diameter and length of harvested deer in the output layer represent the two output neurons in the MLPANN model. The MLPANN can have more than one hidden layer; however, theoretical works have shown that a single hidden layer is sufficient for an ANN to approximate any complex nonlinear function [34, 35]. Therefore, in this study, a one-hidden-layer MLPANN is used by including the number of neurons between 1 and 30.

As presented in Fig 1, the input layer distributes the input signal *x*_*i*_ (the hunting year and month, age and field-dressed weight of deer) to neurons in the hidden layer. Each neuron *j* in the hidden layer sums up its input signals *x*_*i*_ after weighting them with the strengths of the respective connections *w*_1*ji*_ from the input layer and computes its output *z* (Eq 2) as a tangent sigmoid transfer function of the sum, given by

$$z=\frac{e^{\sum w_{1ji}x_{i}}-e^{-\sum w_{1ji}x_{i}}}{e^{\sum w_{1ji}x_{i}}+e^{-\sum w_{1ji}x_{i}}}$$ (2)

The output (antler beam diameter and length of WTD) of neurons in the output layer is computed in the same manner by using linear transfer function. Following this calculation, a learning algorithm is used to adjust the strengths of the connections to allow a network to achieve a desired overall behavior.

#### Metrics for evaluating model performance

Comparisons of expected observations against observed observations provides a valuable guide to overall model performance. The performance of models can be evaluated relative to past observations, relative to other models or against our own theoretical expectations. Agreement with observations is inherently partial. Models agree with some observations but not all. A model can certainly perform well against historic observations, and the precision and accuracy of the fit can be quantified [36].

Chang, Hanna [37], Yu et al. [38] and US EPA [39] used and recommended Mean Absolute Error (*MAE*), Root Mean Squared Error (*RMSE*), Fraction of Model Predictions (*FACT2*), Pearson Correlation Coefficient (*r*) and Index of Agreement (*IA*) to quantify the differences between model predictions and observations and to assess the effectiveness of each model to make precise predictions.

*MAE* and *RMSE* provide a good indication of how close the predicted and observed values are [40, 41] and are calculated as follow:

$$MAE=\frac{\sum_{i=1}^{n}\left(|{\hat{Y}}_{i}-Y_{i}|\right)}{n}$$ (3)

and

$$RMSE=\sqrt{\frac{\sum_{i=1}^{n}{\left({\hat{Y}}_{i}-Y_{i}\right)}^{2}}{n}},$$ (4)

where *Y*_*i*_ is the observed value of antler beam diameter or length for the *i*^*th*^ WTD, ${\hat{Y}}_{i}$ is the predicted value of antler beam diameter or length for the *i*^*th*^ WTD and *n* is the number of observations in the dataset. Smaller values of *MAE* and *RMSE* indicate smaller error, which shows better agreement between predicted and observed values.

*FACT2* that satisfy

$$0.5\leq \frac{{\hat{Y}}_{i}}{Y_{i}}\leq 2.0$$ (5)

is another good indicator of the agreement between predicted and observed values [40, 41]. A scatter plot with the 1:1 correspondence line, together with the 1:2 and 1:1⁄2 lines allows a quantitative comparison between predictions and observations. A count of the fraction of points within 1⁄2 and 2 times the observations, *FACT2*, is a useful metric for model evaluation. *FACT2* values closer to one indicate closer match between predicted and observed values and thus indicate better model performance.

*r* is a measure of the strength and direction of a linear relationship between two variables. A correlation coefficient of 1 indicates a perfect one-to-one linear relationship and -1 indicates a negative relationship. The calculation of *r* is below:

$$r=\frac{1}{n-1}\sum_{i=1}^{n}\left(\frac{{\hat{Y}}_{i}-\bar{\hat{Y}}}{\sigma_{\hat{Y}}})(\frac{Y_{i}-\bar{Y}}{\sigma_{Y}}\right)∙$$ (6)

In model comparison, *r* value provides a measure of deviation between predicted and observed values and is expected to be positive.

*IA* is given by Willmott et al. [42]:

$$IA=1-\frac{\sum_{i=1}^{n}{\left({\hat{Y}}_{i}-Y_{i}\right)}^{2}}{\sum_{i=1}^{n}{\left(\left|{\hat{Y}}_{i}-\bar{Y}|+|Y_{i}-\bar{Y}\right|\right)}^{2}}∙$$ (7)

*IA* is a nondimensional measure and bounded by 0 and 1. The value of *IA* closer to 1 indicates better agreement between predicted and observed values and better model performance.

## Results and discussion

This study presents the assessment of linear model and MLPANN model with three different learning algorithms (L-M, BR and SCG) to predict antler beam diameter and length from the WTD harvest dataset obtained from the BCWMA. Results were compared to determine the best model and the best learning algorithm of MLPANN model for the analysis of WTD harvest dataset.

In the analyses of antler beam diameter and length from the training dataset using linear models, four principal assumptions of linearity, independence, Normal distribution and equal variance of residuals were provided for the inferences on factors affecting the two antler measures. Then, univariate linear model analyses of antler beam diameter and length were completed to better understand the statistical significance of the model in Eq 1 and predictor variables in the model. The results from linear model analyses indicated that all factors were significant (p<0.05). Estimates of regression coefficients of age (${\hat{\beta }}_{1}=5.49$ and 4.36) and field-dressed weight (${\hat{\beta }}_{2}=0.88$ and 0.75) in Eq 1 showed that there were positive linear relationships between the effects of age and field-dressed weight and the traits of antler beam diameter and length. This result is consistent with previous reports [6, 7, 43]. Our results also showed that age is related to antler characteristics and body size in WTD. The implication of this in deer ecology or hunt management is that the age of the deer or the weight of the deer can be predicted from the antler size characteristics and thus providing the wildlife managers extra information needed to make herd health decisions. Specifically, antler traits reflect male quality or affect male-male competition which has implications for sexual selection. Deer herd managers equipped with this type of information could encourage the hunting of males with bigger antlers to prevent directional selection in the herd. Results also demonstrated *r*^2^ (coefficient of determination) values of 81.53% and 80.29%, respectively, suggesting a large portion of variation in antler beam diameter and length was interpreted by the linear model.

In the analyses of antler beam diameter and length from training dataset to develop ANN model structure, the network was defined as a one-hidden-layer MLPANN. Learning in one-hidden-layer MLPANN was made using one of the back propagation (L-M, BR or SCG) algorithms with the number of neurons between 1 and 30. Determining the number of neurons in the hidden layer is an important task in the network [44, 45] since the convergence rate of the network and the time elapsed for prediction may be affected by the number of neurons in the hidden layer [46].

**Fig 2:**
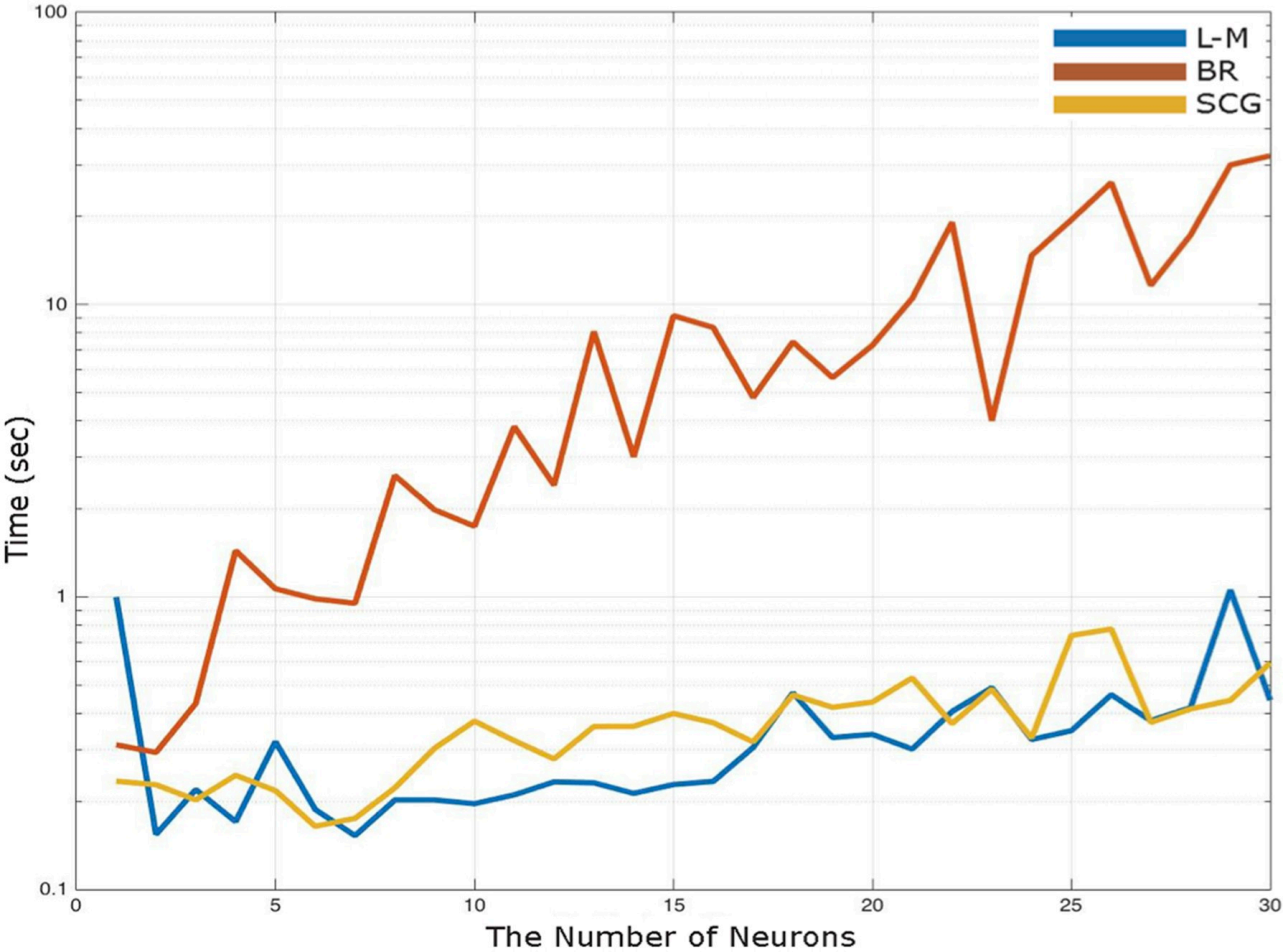
Effect of the number of neurons on the performance of Levenberg–Marquardt (L-M), Bayesian Regularization (BR) and Scaled Conjugate Gradient (SCG) learning algorithms in the Multi-Layer Perceptron Artificial Neural Network (MLPANN) model.

Fig 2 shows the effect of the number of neurons on the time elapsed to learn the structure of network by algorithms (L-M, BR and SCG). As presented in Fig 2, there was a positive linear relationship between the elapsed time for prediction and the number of neurons within L-M, BR and SCG learning algorithms. The learning algorithms of L-M and SCG had similar elapsed time in MLPANN model; however; BR learning algorithm spend much more time to learn the structure in MLPANN model for antler beam diameter and length.

**Fig 3:**
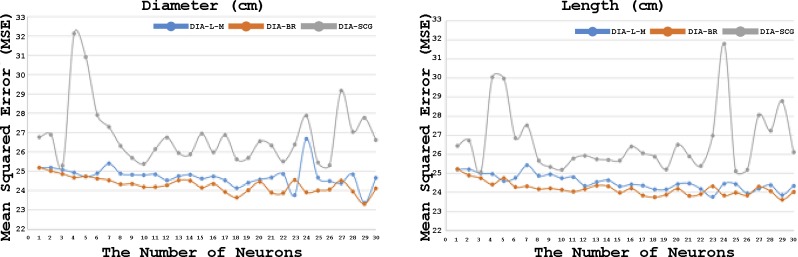
Mean squared error based on the number of neurons using Levenberg–Marquardt (L-M), Bayesian Regularization (BR) and Scaled Conjugate Gradient (SCG) learning algorithms in the Multi-Layer Perceptron Artificial Neural Network (MLPANN) model for antler beam diameter and length in training dataset.

The optimum number of neurons was determined based on the minimum value of mean squared errors obtained by comparing the predicted values with the observed values of antler beam diameter and length for the training dataset (Fig 3). Fig 3 indicated that 29 neurons for L-M and BR algorithms and 3 neurons for SCG algorithm resulted in smallest mean squared errors and the optimized MLPANN structures of 4-29-2, 4-29-2 and 4-3-2 were determined for L-M, BR and SCG algorithms, which had four inputs (hunting year, month, age, field dressed weight) at input layer, the tangent sigmoid transfer function at hidden layer with neurons and two linear transfer functions for antler beam diameter and length at output layer.

**Fig 4:**
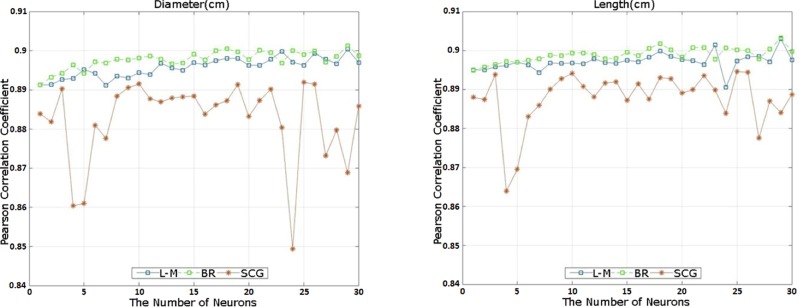
Pearson correlation coefficients between observed and predicted antler beam diameter and length based on the number of neurons using Levenberg–Marquardt (L-M), Bayesian Regularization (BR) and Scaled Conjugate Gradient (SCG) learning algorithms in the Multi-Layer Perceptron Artificial Neural Network (MLPANN) model for antler beam diameter and length in training dataset.

Pearson correlation coefficient based on the number of neurons was calculated to determine the performance of L-M, BR and SCG learning algorithms in the MLPANN model for antler beam diameter and length in training dataset (Fig 4). Correlation coefficients ranged between 0.85 and 0.91 with L-M and BR learning algorithms resulting in similar values compared to those from SCG learning algorithm across the number of neurons. The highest correlation coefficients were obtained from the optimized MLPANN structures of 4-29-2, 4-29-2 and 4-3-2 for L-M, BR and SCG algorithms (Fig 4).

### Comparison of linear model and MLPANN model with L-M, BR and SCG algorithms

Linear model and MLPANN model with L-M, BR and SCG learning algorithms were developed by using training dataset. The performance of the models was determined quantitatively in terms of *MAE*, *RMSE*, *FACT2*, *r* and *IA* after analyzing antler beam diameter and length from test dataset using the models and metrics for model performance (Table 3). As seen in Table 3, metrics for linear and MLPANN models were similar within antler beam diameter and length. However, metrics values of *MAE* and *RMSE* for linear model and the MLPANN model with BR learning algorithm indicated smaller error and lower deviation relative to the mean values of antler beam diameter and length in comparison other MLPANN models demonstrating better agreement of the predicted and observed values of antler beam diameter and length. However, MLPANN model with SCG learning algorithm resulted in the highest error within the models.

**Table 3:** Metrics for model performance using antler beam diameter and length from test dataset.

Model Evaluation Parameters	Diameter (cm)				Length (cm)			
	Linear Model	ANN Model			Linear Model	ANN Model		
		L-M	BR	SCG		L-M	BR	SCG
*MAE*	6.16	6.20	6.14	6.19	4.46	4.42	4.36	4.53
*RMSE*	8.12	8.25	8.19	8.26	5.66	5.56	5.49	5.67
*FACT2*	0.98	0.99	0.99	0.99	0.95	0.94	0.94	0.94
*r*	0.80	0.80	0.80	0.79	0.83	0.84	0.84	0.83
*IA*	0.87	0.86	0.87	0.86	0.90	0.90	0.90	0.90

ANN = Artificial Neural Networks, L-M = Levenberg–Marquardt, BR = Bayesian Regularization, SCG = Scaled Conjugate Gradient, MAE = Mean Absolute Error, RMSE = Root Mean Squared Error, FACT2 = Fraction of Model Predictions, r = Pearson Correlation Coefficient, IA = Index of Agreement.

**Fig 5:**
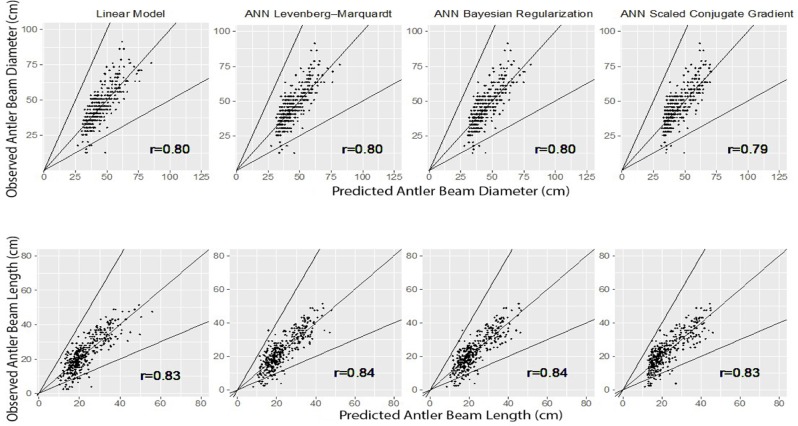
Predicted versus observed antler beam diameter and length for test dataset by Linear model and Levenberg–Marquardt (L-M), Bayesian Regularization (BR) and Scaled Conjugate Gradient (SCG) learning algorithms in the Artificial Neural Network (ANN) model.

The scatter plots of predicted versus observed values are displayed in Fig 5. A 1:1 line is added on each graph to facilitate the comparison to the ideal model, and a factor of two scatter is indicated by the dashed 1:2 and 2:1 lines [47–49]. As shown in Fig 5, all the models seem to over-predict lower values. *FACT2* and *IA* metric values were between 0.98 and 0.99, between 0.86 and 0.87 for antler beam diameter and between 0.94 and 0.95, equal to 0.90 for antler beam length, respectively. These metric results indicated that all models resulted in close match and good agreement between predicted and observed values and thus good performance for all models.

The Pearson correlation coefficients are also added on the graphs to measure the strength of the linear relationship between the predicted and observed values [38]. The metric values of *r* were between 0.79 and 0.80 for antler beam diameter and between 0.83 and 0.84 for antler beam length. The *r*^*2*^ values demonstrated that 63.04% to 64.32% and from 69.22% to 71.23% of the total variation in the antler beam diameter and length could be interpreted by the respective models.

Higher values of *FACT2*, *r* and *IA* were obtained from the MLPANN model with BR learning algorithm and linear model which indicated better agreement of the predicted and observed values of antler beam diameter and length. In this study, overall the MLPANN model with BR learning algorithm provided more accurate results compared to the other MLPANN models in predicting antler beam diameter and length. The utilization of the ANN statistical methodology may provide wildlife managers another tool in the evaluation of data to assist in the development of appropriate deer management plans. To the best of our knowledge this is the first manuscript that reported the application of ANN to deer harvest data and also demonstrated the potential utility of ANN to better understanding antler characteristics for use in management decisions.

## Conclusion

From the results and discussion above it can be concluded that there is a linear relationship between field-dressed weight, age of the animal, and antler characteristics. This knowledge will be very useful in making management decision. in deer herd in the WMA Based on various performance criteria (*MAE*, *RMSE*, *FACT2*, *r* and *IA*), ANN model with BR backpropagation learning algorithm performed similarly when compared to ANN models with L-M and SCG backpropagation learning algorithms in predicting the antler beam diameter and length of WTD as outputs in this study. It is important to underline the potential of ANN models with L-M, BR and SCG backpropagation learning algorithms for mapping non-linear relationship between input and output variables. However, comparison of results from linear model and ANN models with learning algorithms indicated that all models yielded good fit with the antler beam diameter and length of WTD in training and test dataset. However, these results underscore the fact that ANNs can compete with linear models for the modeling of the antler beam diameter and length of WTD and thereby expanding the statistical tool kit available to analyze data and make predictions in herd management.

## Supporting information

S1 Appendix Excel spreadsheet containing raw data from the study. Each sheet contains the individual data points used in training and test process.
(XLSX)

S2 Appendix R and MATLAB scripts. Script contains the R and MATLAB programs for the analysis of the training and test datasets. (R)
(R) Click here for additional data file.
